# Tricuspid Regurgitation Complicating Heart Failure: A Novel Clinical Entity

**DOI:** 10.31083/j.rcm2509330

**Published:** 2024-09-18

**Authors:** Rongyang Xi, Muhammad Ahsan Mumtaz, Dingli Xu, Qingchun Zeng

**Affiliations:** ^1^The First School of Clinical Medicine, Nanfang Hospital, Southern Medical University, 510515 Guangzhou, Guangdong, China; ^2^Department of Cardiology, State Key Laboratory of Organ Failure Research, Nanfang Hospital, Southern Medical University, 510515 Guangzhou, Guangdong, China; ^3^Guangdong Provincial Key Laboratory of Cardiac Function and Microcirculation, Southern Medical University, 510515 Guangzhou, Guangdong, China

**Keywords:** tricuspid regurgitation, heart failure, diagnostic imaging, transcatheter interventions, epidemiology

## Abstract

With the escalating incidence of heart failure, accurate diagnosis is paramount for tailored therapeutic interventions. The tricuspid valve, particularly tricuspid regurgitation, once relegated as the “forgotten valve”, has gained prominence due to increasing evidence implicating severe tricuspid valve disease in the prognosis of diverse cardiovascular conditions. This review delineates recent significant advancements in imaging modalities, transcatheter interventions, and epidemiological and pathophysiological insights regarding tricuspid regurgitation complicating heart failure. A comprehensive understanding of these innovative concepts and technologies can significantly improve patient outcomes.

## 1. Introduction

Heart failure (HF) represents the final stage in the progression of various cardiac conditions and stands as the primary cause of mortality among individuals with cardiovascular diseases. The current aging trend in the global population is contributing to a steady increase in the prevalence and fatality rates of HF, affecting an estimated 64 million patients worldwide and causing an enormous burden to both individuals and society [1, 2, 3]. Among the factors contributing to HF, heart valve diseases resulting from abnormal anatomical structure or function of heart valves play a significant role. While historically overlooked, tricuspid valve disease has recently gained recognition for its impact on the prognosis of HF patients, prompting intensified research efforts to treat this type of valve disease [4, 5, 6, 7]. Tricuspid valve disease is primarily characterized by tricuspid regurgitation (TR); tricuspid stenosis (TS) is rare. Diagnosing and treating TR in conjunction with HF poses considerable challenges in the cardiovascular field. Severe TR disease often precipitates right heart failure, yet accurately assessing the size and function of the right heart proves challenging due to its complex anatomy. Nevertheless, determining the pathogenesis of TR and evaluating the severity of right heart remodeling is crucial for devising optimal treatment strategies. While pharmacological interventions have limitations in halting cardiac remodeling, advancements in imaging technology have enabled more precise measurements of right heart dimensions and function. Additionally, emerging medications and transcatheter valve interventions have shown promise in improving survival and enhancing the quality of life for patients suffering from HF-complicated TR, thereby attenuating HF progression.

## 2. Epidemiology of Tricuspid Regurgitation Complicated by Heart Failure

**Fig. 1.**
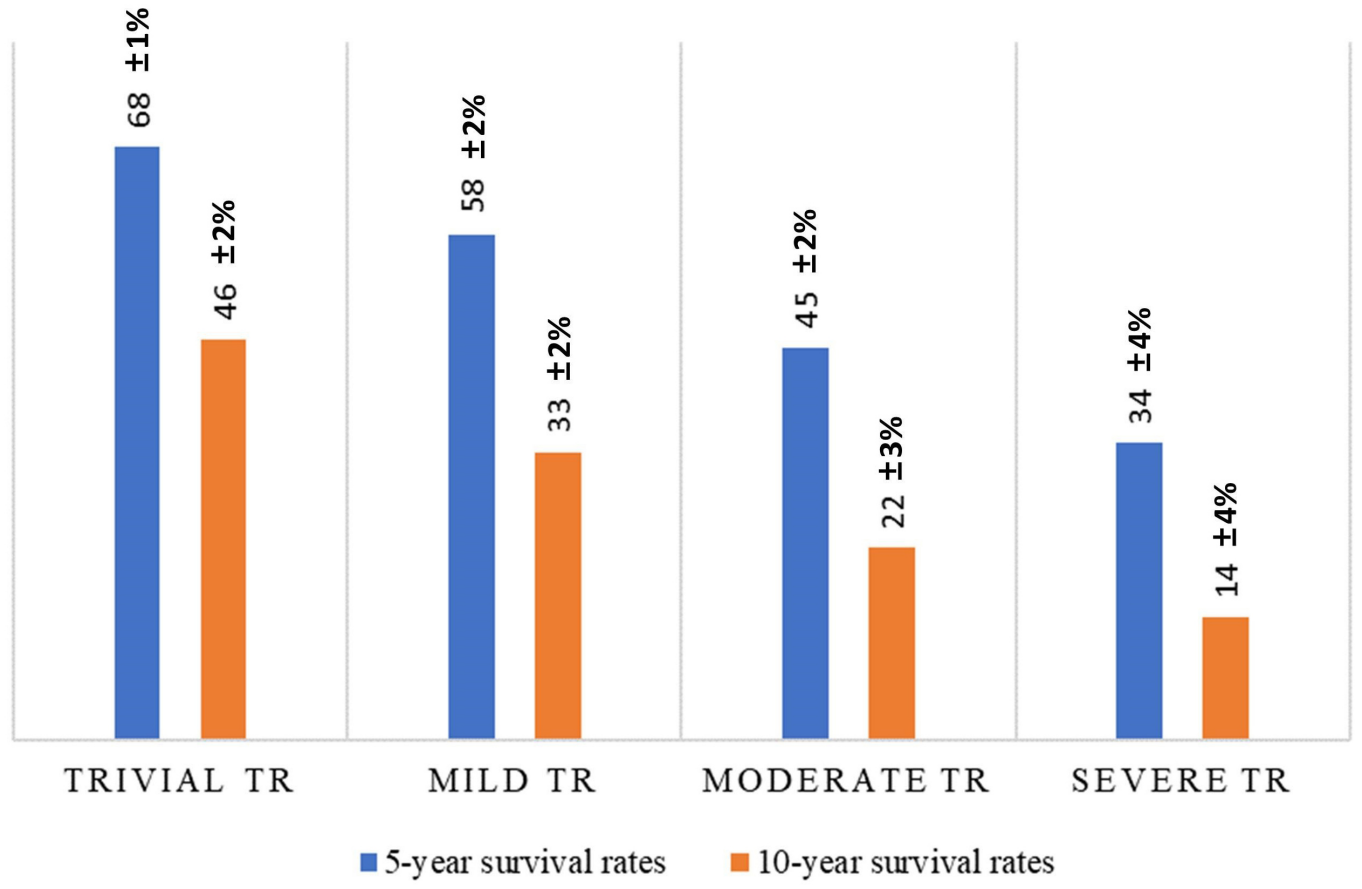
**The relations between tricuspid regurgitation (TR) grades and survival rates under medical management**.

In a UK national cohort study involving 79,043 patients with suspected heart failure who underwent echocardiography, 14.1% exhibited moderate or severe valvular disease, with TR being the most prevalent pathology, accounting for 4.97% of cases [8]. A cohort study of 13,026 patients with Stage B–C HF and <50% left ventricular ejection fraction (LVEF) demonstrated TR in 88% of patients, with the severity of TR correlating with decreased 5-year survival rates (68 ± 1%, 58 ± 2%, 45 ± 2%, and 34 ± 4% for trivial, mild, moderate, and severe TR, respectively) and 10-year survival rates (46 ± 2%, 33 ± 2%, 22 ± 3%, and 14 ± 4% for trivial, mild, moderate, and severe TR, respectively) (Fig. 1), underscoring the prognostic significance of TR in HF progression [9].

Furthermore, a large-scale study involving over 400,000 U.S. HF patients demonstrated a strong association between TR and increased mortality risk across all subgroups. Compared to patients without TR at baseline, those with moderate and severe TR exhibited adjusted risk ratios for mortality of 1.17 (95% confidence interval (CI) 1.14–1.20) and 1.34 (95% CI 1.28–1.39), respectively [10]. A stratified cohort analysis by Barker *et al*. [11] encompassing 33,686 patients revealed varying degrees of TR complexity. TR complicated by HF showed the highest mean Elixhauser comorbidity index (ECI) score, indicating greater disease burden and healthcare resource utilization. In addition, a smaller study involving 220 HF patients concluded that uncorrected tricuspid regurgitation was associated with increased hospitalizations for HF and higher mortality rates [12].

## 3. New Classification of Tricuspid Regurgitation Etiologies

Understanding the diverse etiologies underlying tricuspid valve disease is essential for effective intervention and treatment to mitigate the risk of HF. Previous classifications categorized TR as either secondary to pulmonary hypertension and right ventricular dysfunction or as primary due to isolated right ventricular dysfunction or damage from pacemaker leads [13, 14, 15]. However, recent studies have introduced a more nuanced classification system based on detailed assessments of tricuspid valve (TV) leaflet morphology, the right atrium (RA), and the right ventricle (RV). This new classification delineates TR into three main categories: primary (5–10% of cases), secondary (approximately 80% of cases), and TR related to cardiac implantable electronic device leads (CIEDs) (10–15% of cases) [16, 17, 18].

Primary TR arises from abnormalities in tricuspid valve leaflets, which may stem from various causes such as rheumatic heart disease, carcinoid heart disease, tricuspid valve prolapse, endocarditis, trauma, or congenital heart disease. Among them, Ebstein malformation, carcinoid heart disease (CHD), and drug use–associated infective endocarditis (DUA-IE) are relatively common in clinical practice. In Ebstein malformations, the septal and posterior leaflets of the tricuspid valve are displaced toward the apical portion of the right ventricle. Consequently, the anterior leaflets are restricted to varying degrees, contributing to the different severities of tricuspid regurgitation. In more than 50% of patients, there is a combination of patent foramen ovale or atrial septal defects and abnormal atrioventricular conduction pathway, pulmonary stenosis, and ventricular septal defects. The underlying cause of CHD, which occurs in 20–50% of patients with carcinoid syndrome, may be chronic exposure of valve tissue to high levels of 5-hydroxytryptamine [18]. CHD mainly impacts the right heart valves, leading to tricuspid and pulmonic regurgitation. Tricuspid valve disease is present in over 95% of patients with valvular involvement, and about 90% have moderate or severe tricuspid regurgitation. The treatment focuses on controlling the underlying carcinoid syndrome, targeting valvular heart disease, and managing consequent heart failure [19]. The incidence of DUA-IE has been increasing rapidly over the last decade, whereby 90% of right-sided IE are associated with tricuspid valve disease, and 80% of cases are caused by *Staphylococcus aureus*. A meta-analysis showed that redundant organisms were reduced by more than 50% in 89.2% of patients when AngioVac-assisted excision was used [20].

Secondary TR, accounting for over 90% of severe TR cases, typically presents with normal valve leaflets but exhibits structural changes in the tricuspid annulus or RV, leading to inadequate leaflet alignment [21]. Secondary TR can be further classified into ventricular secondary tricuspid regurgitation (V-STR) and atrial secondary tricuspid regurgitation (A-STR) [17, 18]. V-STR commonly occurs in pulmonary hypertension due to pulmonary heart disease or severe left ventricular disease, resulting in right ventricular dilatation, annular deformation, and regurgitation [22, 23, 24]. A-STR, often underestimated, manifests as a dilated annulus and atria with normal valve leaflets but impaired alignment and is more frequently seen in patients diagnosed with chronic atrial fibrillation (AF) or HF with preserved ejection fraction (HFpEF) [21, 22, 25, 26]. Chronic AF can be a risk factor for the progression of TR. Patients with severe TR are likely to have more persistent AF [27, 28]. Indeed, significant TR was observed in 35% of patients diagnosed with chronic AF. These patients exhibited an enlarged RV, reduced RV free wall longitudinal strain, and decreased tricuspid annular diameter changes prior to significant TR development, suggesting a potential association between RV dysfunction and the onset of TR in chronic AF [29].

With the escalating incidence of heart failure, CIED-related TR is exponentially increasing. Further, CIED-related TR, primarily attributed to valve damage from a pacemaker or intracardiac defibrillator leads, is now separately classified. This category has been linked to lower survival rates and increased heart failure-related adverse events [16, 30]. CIED-related TR is a common cause of acquired TR, occurring in around 38% of cases after lead placement [30]. CIED frequently interacts with the tricuspid valve through heterogeneous mechanisms. CIED-related TR may result from lead impingement on the leaflet without causing injury to the TV apparatus, perforation of the leaflet, or adhesions/interference with the subvalvular apparatus [30, 31, 32]. In a small retrospective study (N = 239), 4-year follow-up data showed that CIED-related TR was associated with lower survival rates (hazard ratio, HR 1.69; 95% CI, 1.02–2.78, *p* = 0.040) and more heart failure-related adverse events (HR 1.64; 95% CI, 1.09–2.48, *p* = 0.019) [15]. In another study, moderate to severe TR occurred in 27% of patients with implanted electronic devices, and the number of hospitalizations for heart failure increased accordingly [31].

## 4. Diagnostic Imaging in Tricuspid Regurgitation Complicated by Heart Failure

Recent advancements in imaging technology have contributed to our understanding of the significance of TR in the progression of right HF, facilitating timely intervention in managing patients suffering from HF. Given the highly variable nature of the TV in terms of the number of leaflet scallops, regurgitant orifice shape, and annular size, echocardiography remains the fundamental diagnostic imaging modality in diagnosing and assessing the severity of tricuspid regurgitation [33]. The European Society of Cardiology (ESC) guidelines advocate for a multiparametric approach to TR assessment, introducing an extended grading scheme (severe (3+), massive (4+), and very severe (5+)) to complement the traditional three-tier scheme (mild (1+), moderate (2+), and severe (3+)) [17].

**Fig. 2.**
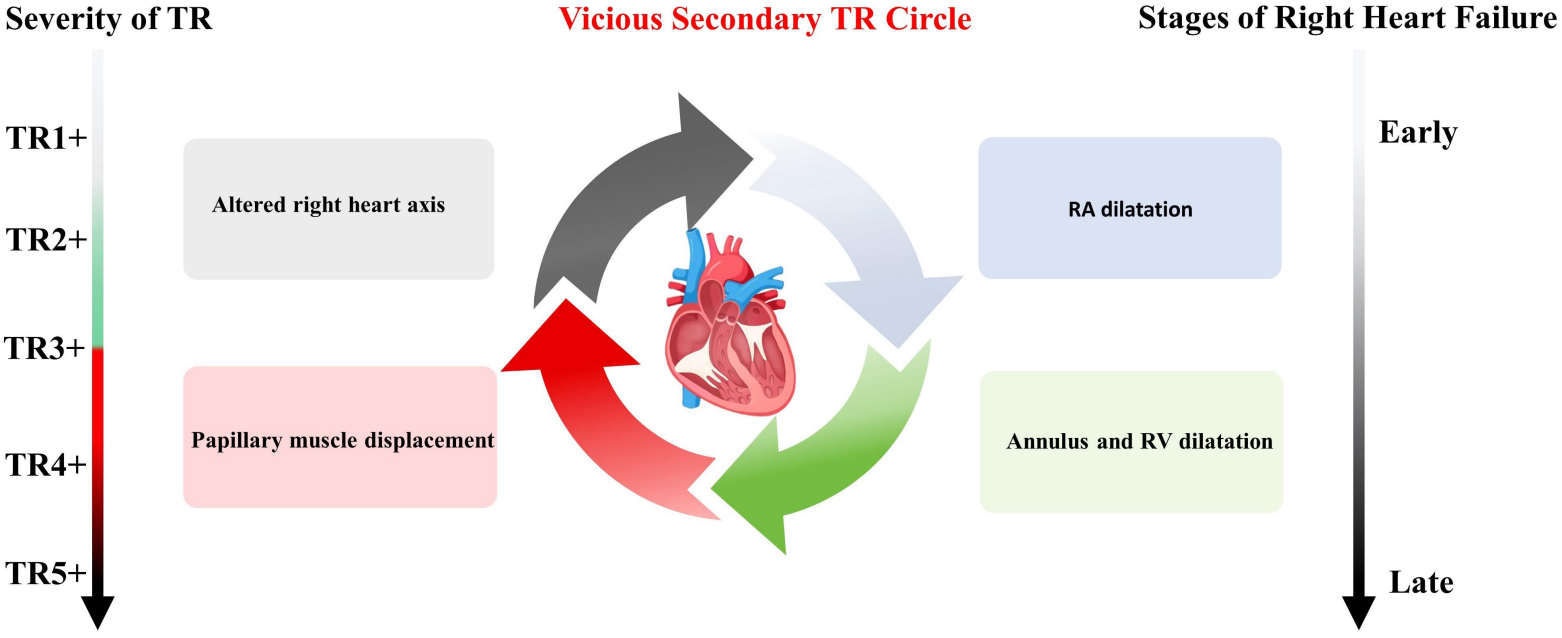
**The detrimental effects of TR on the progression of heart failure**. RA, right atrium; RV, right ventricle; TR, tricuspid regurgitation.

Hahn *et al*. [34] has elaborated on the detrimental effects of TR in the progression of heart failure. Significant TR induces the right heart with volume overload, culminating in dilatation of the right atrium (RA), tricuspid annulus, and right ventricle (RV). The subsequent displacement of the papillary muscle and alteration in the right heart axis exacerbate tricuspid regurgitation, initiating a detrimental cycle marked by progressive dilation and dysfunction of the right ventricle, leading to clinical deterioration (Fig. 2). The proposed parameters for assessing TR and RV functions using echocardiography are presented in Tables 1,2 [34].

**Table 1.** **Severity parameters of tricuspid regurgitation assessment**.

Regurgitation grading	VCW (cm)	PISA measuring by EROA or VCA tracing by TTE (cm^2^)	EROA measuring by Doppler volume calculating methods or VCA tracing by TEE (cm^2^)	Regurgitation volume (mL)	Regurgitation fraction (%)
Mild (1+)	≤0.3	<0.20	-	<30	<30
Moderate (2+)	0.3–0.69	0.20–0.39	-	30–44	30–49
Severe (3+)	0.7–1.39	0.40–0.59	0.75–0.94	≥45	≥50
Massive (4+)	1.4–2.0	0.60–0.79	0.95–1.14	-	-
Torrential (5+)	≥2.1	≥0.80	≥1.15	-	-

VCW, vena contracta width; PISA, proximal isovelocity surface area; EROA, effective regurgitant orifice area; TTE, transthoracic echocardiography; VCA, vena contracta area; TEE, transesophageal echocardiography.

**Table 2.** **Proposed right ventricular function parameters assessment**.

Grade	TAPSE	RV TDI	RV GLS	RV FWS	FAC	3D RVEF
	(mm)	(cm/s)	(%)	(%)	(%)	(%)
Mild dysfunction	14∼17	9∼11	18∼21	20∼23	34∼37	45∼50
Moderate dysfunction	10∼13	6∼8	14∼17	15∼19	30∼33	35∼45
Severe dysfunction	<10	<6	<14	<15	<30	<35

TAPSE, tricuspid annular plane systolic excursion; TDI, tissue Doppler imaging; GLS, global longitudinal strain; FWS, free wall strain; RVEF, right ventricular ejection fraction; FAC, fractional area change; RV, right ventricle; 3D, three dimensional.

Echocardiography encompasses two main modalities: Transthoracic echocardiography (TTE) and transesophageal echocardiography (TEE). However, three-dimensional transthoracic echocardiography (3DTTE) offers a superior assessment of right ventricular volume compared to two dimensional (2D) imaging. This is attributed to its unique ability to capture the short-axis plane of the tricuspid valve, enabling simultaneous visualization of all leaflets throughout the cardiac cycle, along with their attachment to the tricuspid annulus [35, 36].

TEE, performed at various levels and angles, is the primary imaging modality for guiding transcatheter interventions for tricuspid valve disease. It facilitates valve release and anchoring assessment and provides immediate efficacy during valve placement. The American Society of Echocardiography provides detailed TEE imaging protocols for tricuspid valve assessment, emphasizing multiplanar imaging from different esophageal windows to optimize visualization of the tricuspid valve and surrounding structures [37, 38].

Recently, Liu Y *et al*. [39] outlined the procedure for implanting the LuX-Valve Plus, an innovative transcatheter tricuspid valve replacement device with distinct characteristics, under the guidance of 2DTEE and 3DTEE. They concluded that 2DTEE offers better spatial and temporal resolution for precise anatomical monitoring, such as confirming leaflet engagement. However, 3DTEE is preferable for real-time imaging of the entire device, particularly to ensure proper valve leaflet opening [39].

Echocardiography aids in clarifying the TR etiology and severity, which is primarily assessed through TTE. If a discrepancy is observed between the clinical presentation and echocardiographic findings, particularly in symptomatic patients, invasive hemodynamic testing via catheterization may be considered [36].

TEE guidance is often interfered with by shadowing through foreign bodies, such as leads or mechanical prostheses. In such cases, intracardiac echocardiography (ICE) is increasingly employed as a complementary or alternative imaging modality to guide transcatheter tricuspid valve therapy. ICE is an adjunct to TEE, offering high-resolution images and avoiding artifacts on the left side of the heart, thus enhancing imaging quality and precision [40]. Recently, Davidson *et al*. [41] reported a systematic application of four-dimensional intracardiac echocardiography (4D-ICE) as an intraoperative imaging modality during transcatheter annuloplasty. This study demonstrated improved visualization with 4D-ICE compared to TEE, highlighting the potential of ICE for innovation and advancement in transcatheter TV repair procedures [41].

As tricuspid regurgitation progresses to the end stages, patients often develop symptoms of heart failure. When assessing cardiac function on the right side and quantifying tricuspid regurgitation severity, cardiac magnetic resonance (CMR) imaging is particularly useful. A seminal study conducted by Yang Zhan *et al*. [42] employed CMR to evaluate the autonomous prognostic impact of functional TR. Their findings demonstrated that a regurgitant flow of at least 45 mL or a regurgitant fraction of not less than 50% identified patients with the highest mortality risk, even after accounting for clinical and imaging covariates, including right ventricular ejection fraction [42].

Due to its excellent spatial resolution, CMR is particularly valuable when echocardiographic assessment is suboptimal or when the echocardiographic grading of TR does not align with the patient’s clinical presentation. In such scenarios, CMR should be incorporated alongside 3D echocardiography to conduct comprehensive anatomical and functional analyses of the tricuspid annulus and right ventricle and to quantify cardiac remodeling of the atria and ventricle [43, 44, 45].

Computed tomography (CT) imaging is increasingly important in TR assessment, particularly for transcatheter interventions. CT is a complementary tool to transesophageal echocardiography, facilitating the visualization of valve leaflets and TR mechanisms, quantifying TR severity, and the anatomical measurements to choose the right device [46]. The presence of atrial arrhythmias, intracardiac devices or leads, and chronic kidney disease pose challenges in achieving optimal CT imaging. To achieve high-quality functional reconstruction of CT images, adherence to three key principles is essential: electrocardiogram gating throughout the cardiac cycle, avoiding dose modulation, and utilizing minimal available visual alignment (0.5–0.625 mm) [47]. Additionally, precise control of when to conduct image acquisition and contrast agent injection is critical for obtaining high-quality CT images and optimizing the diagnostic yield.

**Fig. 3.**
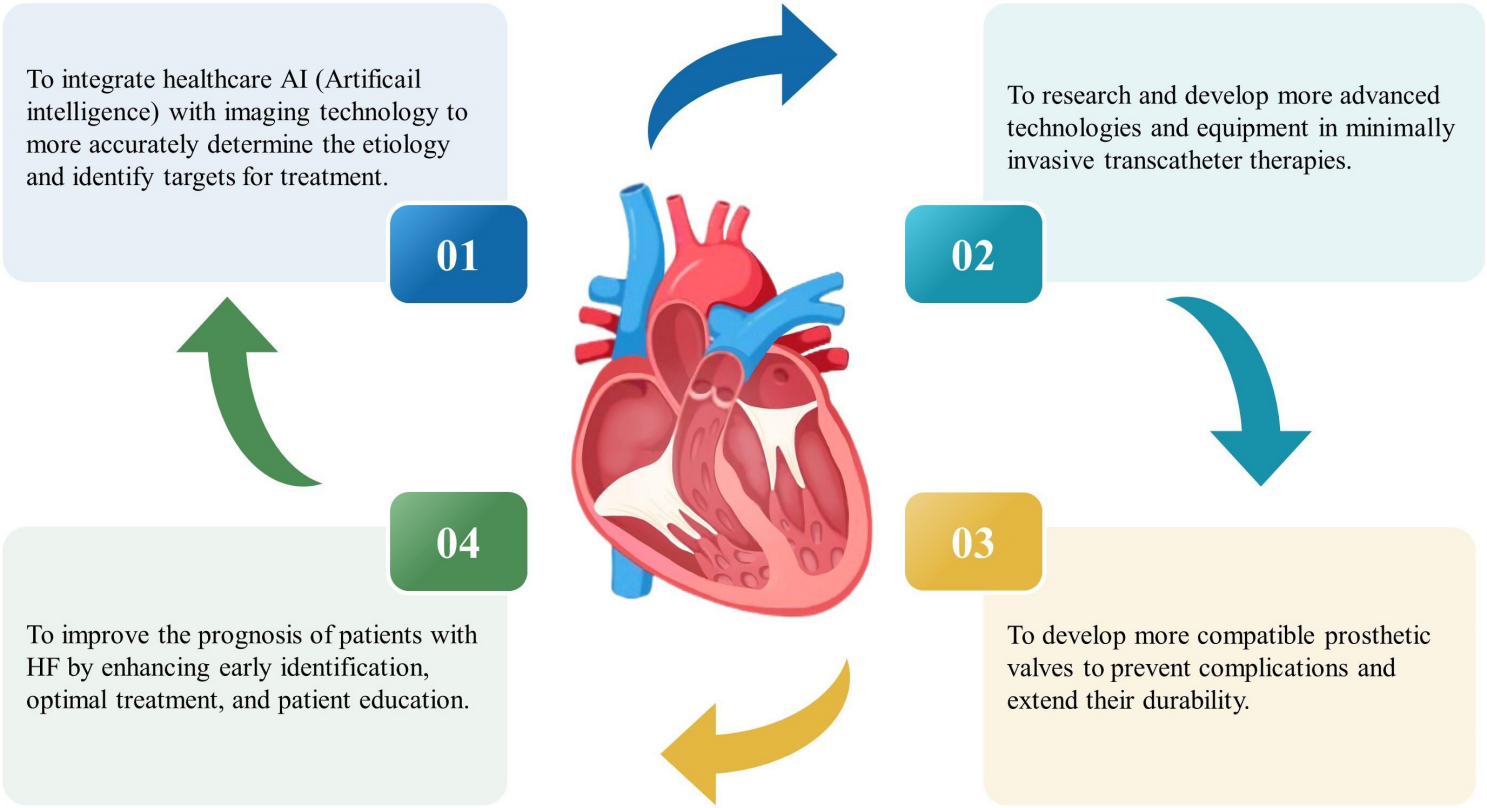
**Future research on the diagnosis and treatment of tricuspid valve disease**. HF, heart failure.

Finally, the use of artificial intelligence (AI) in cardiac imaging is expected to grow in the future. AI technology can improve the accuracy of the cardiac structure and function image segmentation, enabling more accurate quantitative assessment. The emergence of advancements such as a general model for image analysis and AI-assisted diagnostic system indicates that tricuspid disease can be diagnosed by the simplest plain CT in the future, which is projected to determine the etiology of TR and identify potential targets for treatment more accurately (Fig. 3).

## 5. Treatment of Tricuspid Regurgitation Complicated by Heart Failure

The primary manifestation of tricuspid valve disease is the presence of TR. It was widely held that TR was secondary to left-sided heart disease and would resolve following appropriate treatment of the left-sided lesions. As a result, patients with TR are often referred late after multiple diseases and a complex chronic HF state have manifested [48, 49]. Untreated patients with severe TR usually also present with congestive right heart failure, impaired cardiac output, and poor long-term survival [50, 51]. Recent studies have concluded that TR has independent prognostic significance on clinical outcomes, and its treatment options now include both medication and surgical treatments [51].

Medical therapy is mainly based on the use of diuretics to relieve congestion and improve symptoms and is recommended by the ACC/AHA guidelines (Class IIa) for treating TR and right-sided HF [52]. To treat patients with heart failure with reduced ejection fraction (HFrEF) using medications, sodium-glucose cotransporter-2 inhibitors (SGLT2i), renin–angiotensin system inhibitors (ARNI/ACEI/ARB, angiotensin receptor–neprilysin inhibitors/angiotensin-converting enzyme inhibitors/angiotensin receptor blockers), β-blockers, and mineralocorticoid receptor antagonist (MRA) have been added to constitute a new quadruple regimen of guideline-directed medical treatment (GDMT) [53]. Digoxin may be considered in patients with symptomatic HFrEF treated using GDMT (or for those who cannot tolerate GDMT) [16, 54, 55]. The use of digoxin may prove beneficial for patients with secondary ventricular TR due to HFrEF, thereby contributing to a reduction in the hospitalization rate for these individuals with HF. Furthermore, it is suggested that digoxin may offer even greater advantages in patients with advanced HFrEF [54, 55]. In addition, the management of patients with left ventricular dysfunction should include adjunctive pharmacotherapy for the treatment of left heart failure [56]. Treatment of left-sided HF can directly or indirectly improve right ventricular function, and the severity of TR [52, 57] since the right ventricular function can be indirectly improved by decreasing mitral regurgitation, decreasing LV filling pressures, enhancing pulmonary vascular compliance, and decreasing right ventricular afterload. Therefore, GDMT is recommended for patients diagnosed with TR and left-sided heart failure [54, 58]. In patients with HFrEF, each of the four drugs recommended by the guidelines for Class I indications improves left ventricular function, with beta-blockers demonstrating the greatest efficacy [53].

However, medical therapy has a limited role. Data supporting medical therapy in improving the long-term prognosis of these patients are lacking [59]. Surgery has been shown to be effective in improving symptoms of severe TR [36, 60, 61]. The ACC/AHA guidelines, as well as the ESC/EACTS guidelines, both advocate for surgical intervention of TV in patients with severe TR who undergo left-sided valve surgery (Class I recommendation). In addition, both guidelines recommend concomitant tricuspid valve surgery (Class IIa recommendation) for patients undergoing left-sided valve surgery, even if they only have mild to moderate secondary TR and are also presenting with annular dilatation or signs and symptoms of right-sided HF [62].

The surgical procedures for the tricuspid valve can be performed through a right-sided minimally invasive open thoracotomy or a median sternotomy [61]. The TV replacement is only performed in a small proportion of patients, accounting for about 10%–15% of reported cases [63, 64]. Studies have shown that TV repair is more favorable than TV replacement regarding all-cause mortality [65, 66]. TV repair is predominantly performed with an annuloplasty [67]. Currently, the most common annuloplasty technique is an incomplete semirigid prosthetic ring [66, 68]. TV replacement is indicated for tricuspid leaflet anomalies that are not repairable, including carcinoid heart disease, rheumatic heart disease, partial Ebstein’s anomaly, and recurrent tricuspid regurgitation after a previous repair.

Many patients may decline to undergo TV procedures due to high risk or contraindications [69, 70]. Transcatheter interventions for TV are a minimally invasive and comparatively safer alternative to cardiac surgery [71]. The main approaches to transcatheter tricuspid valve intervention (TTVI) are leaflet approximation, annuloplasty repair, heterotopic caval valve implantation (CVI), and transcatheter TV replacement (TTVR) with orthotopic valve implantation [72]. Table 3 provides a summary of several typical devices that are available for TTVI.

**Table 3.** **Available devices for transcatheter tricuspid valve intervention**.

Mechanism	Device name	Manufacturer	Indications and main features
Leaflet approximation	TriClip	Abbott Vascular	(1) To restore coaptation and reduce regurgitant orifice area
	PASCAL	Edwards Lifesciences	(2) Most frequently used among tricuspid devices
			(3) CE-mark approved technique
Annuloplasty	Cardioband	Edwards Lifesciences	Useful for regurgitation caused by annular dilatation and is effective in atrial functional TR
Heterotopic valve replacement	TricValve	Products + Features	(1) Reduction in the venous congestion and backflow associated with TR
	Tricento	New Valve Technology	(2) Useful for severe annular dilatation and those with large coaptation gaps with no option for direct valve treatment
Orthotopic valve replacement	NaviGate	NaviGate Cardiac Structures	(1) Implanting a prosthetic valve in the tricuspid annulus
	EVOQUE	Edwards Lifesciences	(2) Completely resolving residual TR
	Lux-Valve	Jenscare Biotechnology	
	Lux-Valve Plus	Jenscare Biotechnology	
	Intrepid system	Medtronic	
	Trisol system	Trisol Medical	
Leaflet repair and orthotopic valve implantation	CroiValve DUO	CroiValve	(1) A novel approach combining repair and replacement
			(2) Suitable for a wide range of patients

CE, Conformité Européenne; TR, tricuspid regurgitation.

The TTVI transcatheter edge-to-edge repair (TEER) uses an approach that holds the tricuspid leaflets together by placing clips, which increases the coaptation of the tricuspid leaflets and reduces regurgitation; it is one of the most performed techniques to achieve leaflet approximation. In multiple studies, the use of TEER has been demonstrated to be safe and effective for patients suffering from severe TR, improving their overall quality of life [51]. Two TEER devices, TriClip (Abbott Vascular, Santa Clara, CA, USA) and PASCAL (Edwards Life Sciences, Irvine, CA, USA), have been Conformité Européenne (CE)-marked. Tricuspid transcatheter edge-to-edge repair (T-TEER) using the TriClip or leaflet approximation with the PASCAL systems is the most frequently used system for percutaneous tricuspid leaflet repair [33]. The initial findings of TEER utilizing both TriClip and PASCAL therapies have shown promising outcomes (Table 4, Ref. [51, 73, 74, 75, 76]).

**Table 4.** **Devices and study outcomes**.

	Coaptation		Annuloplasty	Replacement	
	TriClip	PASCAL	Cardioband	Heterotopic	Orthotopic
	(n = 175)	(n = 65)	(n = 37)	(n = 35)	(n = 10)
Mechanism	Edge-to-edge clipping	Edge-to-edge clipping with spacer	Direct annuloplasty with cinchable ring and anchors	Valve replacement in IVC ± SVC position	Valve replacement in TV position
Study	TRILUMINATE Pivotal [51]	CLASP TR [73]	TRI-REPAIR and TriBAND [74]	TRICUS EURO [75]	Yuan Z [76]
Baseline characteristics					
NYHA Class III or IV	104	46	24	35	10
EROA, cm^2^	-	0.7	0.8	0.82	-
VC, mm	-	14	15	11.4	-
RVFAC, %	-	36.9	-	47.7	-
TAPSE, mm	17	14	17	18	-
Gap width, mm	-	-	-	-	-
Outcomes					
Technical/procedural success	170/-	59/49	34/31	34/33	10/10
Longest follow-up	1 year	1 year	1 year	6 months	30 days
Mortality	9.40%	10.80%	13.50%	8.50%	0
TR ≤ moderate	88%	86.00%	73%	-	100%

EROA, effective regurgitant orifice area; IVC, inferior vena cava; RVFAC, right ventricular fractional area change; SVC, superior vena cava; TAPSE, tricuspid annular plane systolic excursion; TV, tricuspid valve; VC, vena contracta; NYHA, New York Heart Association; TR, tricuspid regurgitation.

A prospective randomized trial of the TriClip TEER System for managing severe TR (NCT03904147) enrolled 350 patients, who were randomly assigned in a 1:1 ratio to receive either TEER or medical therapy (control group). The TEER group demonstrated superiority over the control group regarding the primary endpoint. Specifically, the incidence of tricuspid regurgitation of no more than moderate severity at day 30 in the TEER and the control groups was 87.0% and 4.8%, respectively (*p* < 0.001). A total of 98.3% of patients in the TEER group had no significant adverse events at 30 days and a reduced TR severity at 1 year of moderate or lower. These results demonstrate that TEER can be safely used for severe TR, effectively reduce TR severity, and improve patients’ quality of life [51]. In another TEER study, TR was diminished in most patients (86%) by at least 1 grade [77].

The PASCAL device was initially developed to manage mitral regurgitation. The PASCAL TV repair device is a 22Fr system with the benefits of leaflet clasping and the physical characteristics of a spacer. Using this technique, some disadvantages observed with other devices can be overcome by eliminating large coaptation gaps and further reducing the total regurgitant area. In a prospective early study (the single-arm, multicenter, prospective CLASP TR early feasibility study) involving 65 patients, there was an obvious reduction in the severity of TR after 1 year (*p* < 0.001). The Kaplan–Meier analysis showed rates of 12.1% for all-cause mortality and 21.5% for heart failure hospitalization. The New York Heart Association (NYHA) functional class exhibited a significant increase (*p* < 0.001), and 92% of patients were in Class I or II. Moreover, there was a substantial improvement of 94 m (*p* = 0.014) in the 6-minute walk distance and an impressive enhancement of 18 points (*p* < 0.001) in the overall Kansas City Cardiomyopathy Questionnaire score. The study demonstrated a low incidence of complications and high survival rates using the PASCAL system, accompanied by significant and consistent improvements in one-year tricuspid regurgitation, functional status, and quality of life. However, experience using this device is limited, and the follow-up duration is short [73].

Annuloplasty addresses annular dilatation by reducing the tricuspid annular diameter, thereby improving leaflet coaptation and decreasing TR. The primary device currently utilized in clinical practice is the Cardioband Tricuspid Valve Repair System (Edwards Lifesciences), which comprises a direct, adjustable, and incomplete surgical nylon ring that is advanced through a transfemoral 24Fr sheath. It is then securely fastened to the atrial side of both the anterior and posterior tricuspid annulus, effectively replicating the outcomes achieved with a small-sized annuloplasty. Implantation of the Cardioband System is currently performed in selected centers worldwide [72]. Results after implantation (TRI-REPAIR and TriBAND studies) showed that at the 6-month and 2-year follow-up, the TR grade was decreased to moderate or lower in more than 70% of the patients, and 80% of the patients had improved symptoms (NYHA Class I/II) [78, 79]. One-year outcomes with the TV repair using the Cardioband System demonstrated improved survival rates, low rehospitalization rates, and good quality of life [74]. However, the significant need for procedural imaging has hindered the application of the Cardioband system. Furthermore, the current limitations of annular devices include excessive annulus dilation, substantial coaptation gaps, and inadequate or retracted septal leaflets [33].

TTVR may emerge as a promising therapeutic option for patients with severe TR who are deemed unsuitable for transcatheter repair due to elevated surgical risk and unfavorable prognosis [80]. Various TTVR systems are currently undergoing preclinical and clinical development and research, which can be classified into two types: Heterotopic (caval valve implantation) and orthotopic TTVR [72]. The heterotopic TV replacement procedure involves strategically placing one or more valves within either or both vena cava to effectively mitigate regurgitation from the venous system, alleviate venous congestion, and relieve symptoms associated with TR. The TricValve (P + F Products + Features), a specialized device for caval valve implantation (CAVI), obtained CE mark approval in 2021. TricValve offers a new treatment option for patients who are not candidates for surgery, patients with very dilated annular gaps or very large junctional gaps, and even patients with pacemakers [75, 81]. Another heterotopic implantation device is the Tricento (NVT, Hechingen, Germany) valve device, which is designed to treat vena cava reflux problems in cases of severe TR without removing the defective tricuspid valve. The Tricento system is a 24Fr transfemoral device featuring a self-expanding stent made of nickel–titanium alloy, which can be customized in length from the superior vena cava (SVC) to just above the hepatic vein. The system can be adapted to existing CIED wires without general anesthesia but requires angiography [82].

Congestion alleviation in symptomatic patients with severe TR can be observed in heterotopic TV replacement. Anatomic and physiologic requirements predict the benefit of these devices [16]. The findings of an observational study demonstrated the safety and efficacy of self-expanding devices placed in both the superior and inferior vena cava for surgical procedures. Notably, significant improvements were observed in patients’ quality of life and NYHA functional classifications at the 6-month follow-up. Nevertheless, no statistically noticeable improvements were noted in hemodynamic parameters or right ventricular volume [75, 83].

Orthotopic valve replacement consists of a catheterized prosthetic valve implanted into the patient’s anatomic TV position, replacing the original diseased TV. Currently, orthotopic transcatheter tricuspid valve replacement is being tested on tricuspid valve leaflets/annulus using various anchoring systems and seems effective in controlling residual TR [84, 85, 86]. However, many uncertainties remain for very large/asymmetric regurgitant orifices, atrioventricular blocks, and tilted or multivalve tricuspid valves, meaning comprehensive imaging trials are needed [47]. Several orthotopic position-switching systems are currently undergoing clinical and preclinical studies. The initial results of the first human trial have already been published using the NaviGate (NaviGate Cardiac Structures), EVOQUE (Edwards Lifesciences), Lux-Valve (Jenscare Biotechnology), as well as Lux-Valve Plus (Jenscare) prostheses [47, 87, 88].

It has been reported that the use of the Gate valve (NaviGate Cardiac Structures) [89], EVOQUE TVR System (Edwards Lifesciences) [88], Lux-Valve (Jenscare) [90], and other devices for orthotopic TTVR surgery, have resulted in good valve performance and significant decreases in residual TR.

The LuX-Valve Plus system, recently reported by Liu Y *et al*. [39], enables three-plane motion coaxial alignment and valve orientation adjustment. The radial force-independent anchoring mechanism ensures stability and safety during valve implantation [39]. Early single-center analyses showed that the LuX-Valve Plus system was safe and effective 30 days after surgery and significantly reduced TR in patients with significant clinical symptoms (Table 4) and complex anatomy [76].

Notably, not all currently published transcatheter TV replacement systems are repositionable. Repositionable designs featured in next-generation devices, such as the Intrepid system (Medtronic, Minneapolis, MN, USA) and the Trisol system (TriSol Medical Ltd., Inc., Yokneam, Israel), have the potential to enhance the probability of successful implantation [91]. The Intrepid is a circular internal stent with a built-in triple-leaflet bovine pericardial valve delivered via a 35Fr delivery system, representing either a transapical or transfemoral channel delivery system. The procedure is also less reliant on imaging as it does not necessitate intricate rotational alignment or reliance on leaflet capture. Theoretically, this should improve implantation success in patients with complicated valve anatomy; a related early feasibility study is underway (NCT04433065) [81]. The Trisol valve consists of a nickel–titanium frame and specially designed dome-shaped valve leaflets, which are held in place by two strips, allowing it to function as a bilobed valve. This novel design facilitates more optimal leaflet closure, thereby preserving right ventricular function after valve replacement. The Trisol valve is axially anchored and can be fitted to any size annulus. The first two Trisol transcatheter tricuspid valve replacement implants were successfully performed in the United States in 2023 [61, 81, 92].

Current challenges in transcatheter TV treatment include difficulty in visualizing the TV on TEE, the presence of a large tricuspid annulus in severe TR, usually associated with a large coaptation gap, insufficient calcification of the valve or annulus, and fragility of the adjacent right coronary artery and valvular tissue. To address these issues, device companies are constantly innovating their prostheses. An example is the CroiValve DUO Triple Cuspid Alignment Transcatheter Valve System (CroiValve, Dublin, Ireland). This system consists of two integrated components. The device utilizes transcatheter implantation technology and is secured to the superior vena cava by a novel anchoring system that leaves the fragile right ventricle and native valve apparatus untouched. The CroiValve valve is securely anchored within the tricuspid valve leaflet using a stent strategically positioned between the native leaflets to minimize the size of the regurgitant orifice and facilitate optimal alignment of the leaflets. The device effectively accommodates anatomical variability and can accommodate very large annular diameters up to 65 mm while avoiding contact with the atrioventricular node and right ventricular (RV) free wall, thereby reducing anatomic contraindications [81]. Theoretically, this device can treat extreme variations in annular anatomy and coaptation gaps. Moreover, the implantation of this system is straightforward, utilizes standard imaging techniques, and is suitable for a wide range of patient populations, including those who are difficult to treat using other valve repair and replacement techniques. Currently, the utilization of this system has been limited in terms of successful cases, and additional data are required to validate its long-term safety and efficacy [93].

As mentioned previously, TV repair is superior to replacement. A meta-analysis of observational and single-arm trials focusing on transcatheter TV repair systems has also demonstrated that cardiovascular complications with edge-to-edge repair incidence are extremely low; however, the residual TR is relatively high [94]. Current data suggest that TTVR may be more effective in completely resolving residual TR. The optimal choice in clinical practice should be determined through a comprehensive evaluation of anatomical and clinical features while carefully considering the pros and cons of repair versus replacement. Furthermore, the durability of prosthetic valves and the occurrence of valve thrombosis after replacement therapy require more long-term follow-up data [95]. Since valve failure occurs in bioprosthetic valves over time, repeat surgery is usually not feasible in patients with multiple comorbidities. The transcatheter valve-in-valve (TVIV) and transcatheter valve-in-ring (TVIR) procedures represent safe and efficacious approaches for repeat surgical interventions. The ESC guidelines also state that in patients with failing tricuspid prosthetic bioprosthetic valves, new transcatheter valve-in-valve implantation may be considered in those at high surgical risk (ESC/EACTS guideline class IIb recommendation, level B evidence), which may reduce TR and improve clinical symptoms [60]. An alternative approach is to investigate more compatible prosthetic valves to prevent complications such as regurgitation and extend their durability (Fig. 3). The current market offers two available valves: The Melody™ valve manufactured by Medtronic, based in Minneapolis, MN, USA, and the SAPIEN3 valve developed by Edwards Lifesciences, headquartered in Irvine, CA, USA. Although long-term data on efficacy remain limited, TVIV and TVIR offer hope to patients with no other viable corrective options [96].

Overall, the current clinical experience and experimental data from TTVI have propelled the ongoing advancement of transcatheter valve therapy. However, larger and more comprehensive randomized studies are imperative to establish definitive clinical and procedural endpoints and outcomes of TTVI, thereby enabling more robust conclusions. The era of percutaneous transcatheter TV interventions is just beginning and will undergo rapid evolution, driven by technological advancements in aortic and mitral valve therapies. These advancements will also prompt research and progress in the surgical treatment for TR.

Finally, the importance of disease awareness and patient education must be addressed in managing chronic diseases, especially HF. Recently, the U.S. National HF Initiative (IMPLEMENT-HF) plans to facilitate incipient identification, optimize therapy, and provide education to enhance the prognosis of patients with HF. In the meantime, the development of the HF Assistant application aims to enable patients to monitor symptoms and medication effectively, share their condition with healthcare providers safely, and establish connections with HF patients.

## 6. Conclusions

In conclusion, managing tricuspid regurgitation complicated by heart failure has significantly evolved over time. Historically, limited understanding and therapeutic options necessitated a shift towards innovative approaches, including developing novel pharmaceuticals and advanced imaging techniques, alongside rapid advancements in minimally invasive transcatheter therapies. These collective endeavors have significantly enhanced the clinical outcomes of patients facing this intricate condition.

Looking ahead, the optimal management of patients with tricuspid regurgitation and heart failure will likely involve a comprehensive approach that integrates the latest imaging modalities, transcatheter interventions and established heart failure medications. Utilizing these diverse strategies can provide renewed optimism and improved results for individuals facing this complex medical situation. It underscores the importance of collaboration among various specialties and the continual exploration of innovative treatments to enhance patient care and quality of life.

## Availability of Data and Materials

All data points generated or analyzed during this study are included in this article and there are no further underlying data necessary to reproduce the results.
